# Six Gynecological Cancer Patients Infected With SARS-CoV-2 After Surgery or Radio-/Chemo-Therapy Treatment: Case Series

**DOI:** 10.3389/fonc.2020.01606

**Published:** 2020-09-15

**Authors:** Chen Liu, Yuhan Huang, Tianyu Qin, Ensong Guo, Peng Wu, Chaoyang Sun, Gang Chen

**Affiliations:** ^1^Department of Obstetrics and Gynecology, Tongji Hospital, Tongji Medical College, Huazhong University of Science and Technology, Wuhan, China

**Keywords:** SARS-CoV-2, COVID-19, gynecological cancer, treatment, suggestions

## Abstract

**Objective:**

Recently, the number of gynecological cancer patients infected with SARS-CoV-2 has been increasing. This article was committed to studying the influence of gynecological tumor treatment history compared to the Coronavirus Disease 2019 (COVID-19), which was of great significance for the treatment of gynecological cancer patients during the outbreak of COVID-19.

**Methods:**

We retrospectively analyzed the diagnosis and treatment of six gynecological cancer patients infected with SARS-CoV-2 in Tongji Hospital in Wuhan from January 30 to March 25, 2020. To better explain the treatment of gynecological cancer patients during the epidemic of COVID-19, we summarized the case characteristics, auxiliary examination, treatment plan, and outcome of these six patients.

**Results:**

We observed a high rate of nosocomial SARS-CoV-2 infection among these six gynecological cancer patients, who were in a low immune state. Also, due to the influence of cancer treatment history, COVID-19-related atypical symptoms became the first symptom of COVID-19 in some cases, which increased the difficulty of diagnosis. Furthermore, in terms of treatment for these cases, immune boosters and reagents that raised white blood cells were applied, except for in symptomatic antiviral treatment. At present, all patients in this study were discharged from the hospital with a good prognosis.

**Conclusion:**

After cancer-related treatment, the gynecological cancer patients became more susceptible to COVID-19. Besides, the history of cancer treatment made the diagnosis of COVID-19 difficult, which also affected the treatment of COVID-19. Therefore, we put forward the corresponding therapy suggestions for gynecological cancer patients during the outbreak of COVID-19.

## Introduction

The Coronavirus Disease 2019 (COVID-19), which was first reported in Wuhan, China, in December 2019, had already evolved into a worldwide crisis (1). So far, the number of patients with newly diagnosed COVID-19 has reached 1,336,378 (by April 7, 2020). The pathogen was identified to be a novel coronavirus named severe acute respiratory syndrome coronavirus 2 (SARS-CoV-2), with a genomic organization similar to the known bat-SL-CoVZC45, bat-SL-CoVZXC21, and SARS-CoV-1 (2, 3). Much the same as the previous two highly pathogenic coronaviruses (SARS-CoV-1 and MERS-CoV2), the newly discovered virus mainly caused a respiratory tract infection through droplet contamination and close contact with those infected (4).

We noticed that cancer patients, with a fragile physical state and a higher chance of exposure during routine follow-up, might require special attention at this stage. Emerging studies suggested that this particular group bore more susceptibility compared to the general population, any infection was prone to develop into a severe event (5) (i.e., requiring admission to ICU, application of mechanical ventilation, or death), and a poor prognosis was likely during disease. Meanwhile, the immunosuppressive condition and possibly more extended incubation period caused by cancer-related treatment posed a severe challenge to clinical decisions. In most cases, patients were advised to delay scheduled chemotherapy, radiotherapy, and targeted therapy to avoid aggravated infection (6, 7). Researchers in Italy recommended a postponement in non-critical cancer treatment. An official French guideline recently released advised cancer patients with COVID-19 to discontinue anticancer treatment (8). ICIs (Immune checkpoint inhibitors), though considered to do less harm to immunocompetence than chemo- and radiotherapy, raised reasonable concern for the possible interplay between ICIs and the coronavirus (9). Yet Zhang et al. reported a case in which a lung cancer patient infected with SARS-CoV-2 recovered from pneumonia while continuing initial targeted therapy (10). Thus, more individualized clinical management should be customized to cope with the complexities of the virus in cancer patients.

Herein we described six COVID-19 patients with gynecologic cancers from Tongji Hospital in Wuhan for the purpose of better understanding the proper medical decision and social support for cancer patients during this pandemic.

## Methods

### Study Design and Patients

We screened six patients with gynecologic cancer who had also been diagnosed with COVID-19 (according to WHO’s official diagnosis) in Tongji Hospital, Huazhong University of Science and Technology from January 30, 2020 to March 25, 2020. We collected data on the clinical characteristics, auxiliary examination, laboratory examination, treatment, and outcome of these six patients. Then, we carried out integrated analysis of these data. Finally, we discovered the characteristics and commonality of diagnosis and treatment of gynecological cancer patients to better influence the treatment of gynecological cancer patients during the COVID-19 epidemic period.

### Data Analysis

To clarify the commonality of diagnosis and treatment of gynecological cancer patients with the SARS-CoV-2 infection, we statistically analyzed the nosocomial infection rate, first symptoms, case characteristics, diagnosis and treatment process, and the outcome of these six gynecologic cancer patients with COVID-19.

## Results

We divided the gynecological patients infected with SARS-CoV-2 in the Tongji Hospital of Wuhan into two categories. Type 1 were gynecological cancer patients with COVID-19 who were undergoing or had just finished cancer radiotherapy, chemotherapy, or immunotherapy, including cases 1, 2, 3, and 4. Type 2 were gynecological cancer patients with COVID-19, who had just undergone cancer surgery, including cases 5 and 6 (Table 1).

**TABLE 1:** Clinical characteristics and treatments of six gynecologic tumor patients with SARS-CoV2 infection.

Patient No.	1	2	3	4	5	6
Type	Type1: gynecological tumor patients with COVID-19 who were undergoing or just finished comprehensive treatment				Type2: gynecological tumor patients with COVID-19, who had just undergone a tumor surgery	
Age	61	66	65	67	27	62
Hospital infection	No	Yes	No	Yes	Yes	Yes
Community infection	Yes	No	Yes	No	No	No
First symptom	Fever,abdominal pain	Fever and chills	Cough, sputum, difficulty breathing	Cough, sputum	Fever, cough, sputum	Fever and chills
COVID-19 related atypical symptoms	−	−	diarrhea and melena	diarrhea	−	atypical viral pneumonia
Time of first symptom	2020/1/4	2020/1/19	2020/2/3	2020/2/11	2020/1/23	2020/2/6
Improvement time	2020/2/16	2020/2/29	2020/3/3	2020/2/20	2020/2/15	2020/2/18
Tumor operation time	2019/6/1	2019/7/1	2019/4/30	2019/9/27	2020/1/21	2020/1/16
The last time of comprehensive treatment of tumor	2019/12/25	2020/1/18	2020/1/6	2020/2/11	−	−
Tumor type	High-grade serous endometrial carcinoma III	High-grade serous endometrial carcinoma IIIc1	Poorly differentiated adenocarcinoma of ovary	Endometrial Carcinoma	Small cell carcinoma of ovary Ic	Poorly differentiated adenocarcinoma of cervix IIB
COVID-19 classification	Common	Common	Severe	Common	Common	Severe
Tests results on hospital admission						
IL-6	17.12	12.4	12.87	< 5	12.93	−
Albumin/globulin	0.63	0.91	1.3	1.93	1.52	0.95
Neutrophil count, 10− cells per L	5.48	0.67	1.05	2.46	5.88	1.85
White blood cell count 10*9 cells per L	6.67	1.91	1.5	3.27	8.84	3.94
Lymph cell count 10*9 cells per L	0.79	1.05	0.34	0.41	2	1.32
Tumor marker	−	−	CA125 11.7	−	CA125 7.3	−
Treatment						
Antiviral	Abidor	−	Abidor	−	Abidor	−
Chinese patent medicine	+	+	+	+	+	+
Anti-infective	+	+	+	−	+	+
Reagents that increase the number of white blood cells	+	+	+	−	−	−
Human albumin reagent	+	−	+	−	−	−
Human globulin reagent	−	+	−	−	+	−
Interferon	+	−	−	−	+	−
Plasma products	−	+	+	−	−	−
Treatment outcome	Alive	Alive	Alive	Alive	Alive	Alive

*–Means not detected or not used rather than missing data.*

Case 1 (61 years old) had received her recent chemotherapy a month before she was infected in her community. The symptoms initially showed as fever, and abdominal pain, which was mistaken for a side effect of chemotherapy, so she did not see a doctor until 15 days later after the fever began. Though she didn’t feel dyspnea, a CT scan showed bilateral pneumonia and ground-glass opacities in both lobes of the lung. Her temperature was up to 39.3°C, with SpO_2_ down to 63% during the disease. After antivirus, Abidor dispersible tablets, and anti-infection treatment for 45 days, this patient was cured of her COVID-19 pneumonia (Table 1). The cancer was shown not to progress by CT scanning.

**FIGURE 1:**
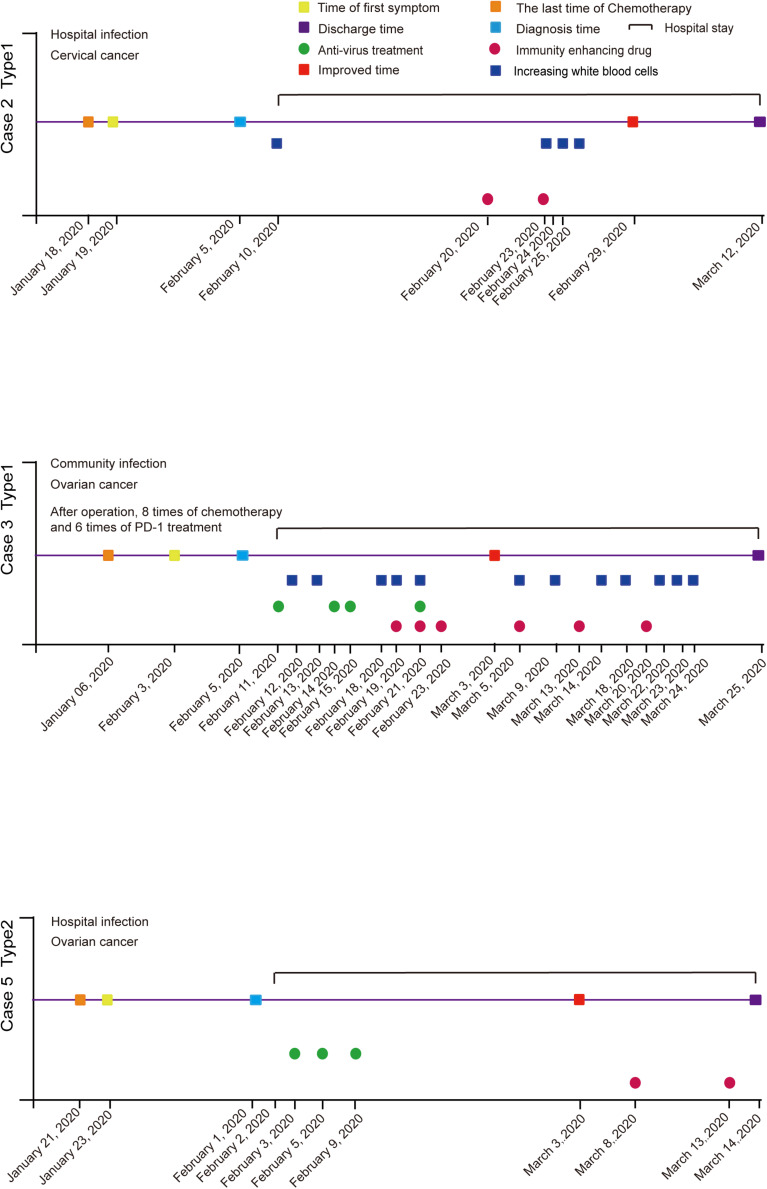
Flow chart of patients’ diagnosis and treatment in gynecological oncology department during the epidemic of COVID-19.

Case 2 (66 years old) had a fever the day after she had finished her chemotherapy in the hospital, which was initially thought to be related to chemotherapy. However, we scheduled a CT scan and PCR test immediately, which was initially negative. But because of the exposure and a chemotherapy-induced bone marrow suppression, she began symptomatic treatment in the hospital. After five days, RT-PCR showed positive in the COVID-19 test. After antivirus and anti-infection treatment, combined with G-CSF (Recombinant Human Granulocyte-stimulating Factor) and immunity enhancing drugs, she was finally discharged from hospital after 35 days (Figure 1).

Case 3 (65 years old) received anti-PD-1 treatment (January 6, 2020) shortly before the infection (February 3, 2020) after a basic operation for ovarian cancer (April 8, 2019). In aggregate, she received eight chemotherapy treatments in combination with six anti-PD-1 treatments. When she complained about chest pain and had been coughing for 7 days, she was diagnosed as a COVID-19 patient. Her lymphocytes, neutrophils, and white blood cells were far below normal on admission. Considering the symptoms, she was classified as a severe patient. Thenceforth, the patient underwent antivirus treatment seven days after her admission (February 19, 2020). The patient was announced critically ill with low serum albumin, human serum albumin was applied several times throughout the course. The patient’s condition began to improve 19 days after admission (March 3, 2020). Subsequent chest CTs showed stable lesions that underwent gradual absorption. Three consecutive RT-PCR tests were negative for SARS-CoV-2. Additionally, cancer markers were within the normal range all the time. At that point, the patient had confirmed a full recovery and was discharged 24 days after admission (March 25, 2020) (Figure 1).

**FIGURE 2:**
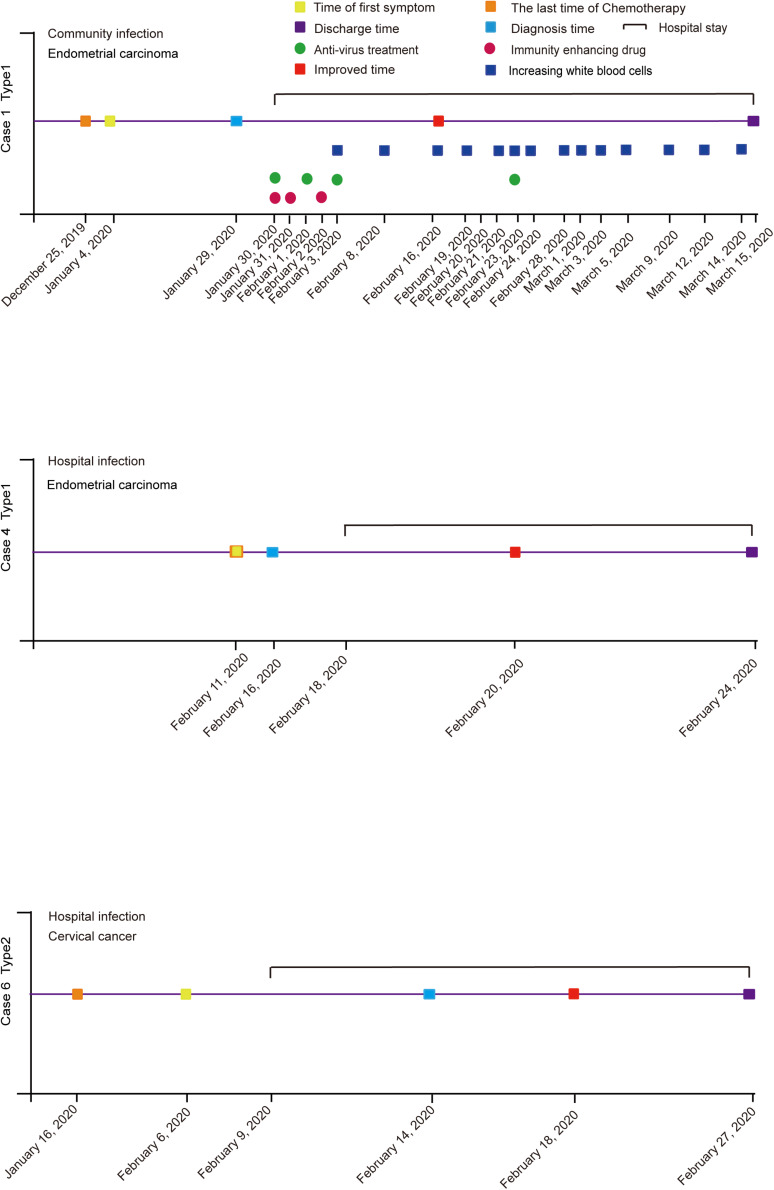
Flow chart of patients’ diagnosis and treatment in gynecological oncology department during the epidemic of COVID-19.

Case 4 (67 years old) began her fever a day after radiotherapy. Her first symptoms included a cough and musty stool as well as the fever. A RT-PCR test was done on the day the fever began, and she was diagnosed as positive for COVID-19 when the result came out. This patient was classified as having a common type of COVID-19 by clinical symptom. After antivirus treatment, she became asymptomatic on day 4. Another three negative RT-PCR tests were done on day 6, which allowed her to return home (Figure 2).

Case 5 was in hospital before people realized what a huge impact COVID-19 would bring. On January 23, 2020, a 27-year-old woman, who had just finished surgery to deal with ovarian cancer a day before, began to have a fever. Doctors did not realize that it was a sign of COVID-19 until other symptoms like a cough and sore throat showed nine days later with a temperature of 38.5°C. After an RT-PCR test, the woman was shown to be positive for the COVID-19 infection. A CT scan showed the lower lobes of both lungs had the shadows of ground-glass opacities (Figure 1).

Case 6 (61 years old) also began a fever in the hospital. She was suffering from a cervical carcinoma and finished her surgery three days earlier. She was visited by a COVID-19 patient before surgery, so when a fever began, a RT-PCR test was quickly applied for the first time. The test confirmed that the fever was because of COVID-19. Although she felt a little dyspnea, CT scanning didn’t show specific characteristics of COVID-19 when she was tested on day 1, while slightly virus-connected changes showed on day 5. She only used some anti-infection drugs combined with non-invasive ventilation treatment. PCR results turned negative three times on the 9th day after diagnosis (Figure 2).

## Discussion

Due to the small number of cases and the incomplete clinical information for these patients, there was a statistical deviation when we concluded. Therefore, the conclusion of this article needs more research to explore our findings fully. According to our observation of these six patients, we found that the influence of cancer treatment on COVID-19 had the following three characteristics.

Firstly, the gynecological cancer patients were susceptible to SARS-CoV-2, they often went in and out of the hospital resulting in an increased chance of exposure to SARS-CoV-2. Cases 2, 4, 5, and 6 were infected by SARS-CoV-2 in the hospital. Case 2 had a fever one day after chemotherapy, case 5’s fever started two days after surgery, case 6 felt chill and a fever developed three days after surgery, and case 4 began to cough just after her radiotherapy. As a study reported, oncology patients had a high nosocomial infection rate (11).

Moreover, cancer patients showed a state of low immunity after surgery or radio-/chemo-therapy treatment, so they became more susceptible to COVID-19 (12). As our results showed, both case 2 and case 3 showed signs of leukopenia; similarly, case 1, case 2, case 3, and case 6 had an inverted ratio of albumin to globulin. A report said that a leukemia patient who had undergone a bone marrow transplant also had a high SARS-CoV-2 infection rate (13). Therefore, according to the progression of cancer, if the cancer was not urgent, it was recommended that patients delay admission, selective surgery, and radiotherapy, with priority, instead, going to treatment with oral chemotherapeutic drugs, endocrine, or immunotherapy to maintain the patient’s condition (8, 14).

Secondly, the history of cancer treatment made the diagnosis of COVID-19 very difficult. The first symptoms of COVID-19, usually fever, were easily confused with the low fever typically exhibited after surgery, cancer radiotherapy and chemotherapy, which increased the difficulty of diagnosis and the timely treatment of cancer patients with COVID-19. Case 5 delayed therapy for nine days because doctors did not identify the cause of her fever.

Besides the confusion over the cause of fever, COVID-19 also presented atypical viral pneumonia increasing the difficulty of confirming a COVID-19 diagnosis. In a case report of a renal transplant patient infected with SARS-CoV-2, it mentioned that the patient’s first symptom was viral enteritis, which was different from COVID-19’s previous reports (15). The CT of case 6 showed COVID-19 related atypical viral pneumonia. Case 3, who had a partial resection of the intestine because of ovarian cancer, had diarrhea and melena, causing severe anemia and hypoproteinemia. Also, she had hypokalemia, hyponatremia, and hypochloremia before the viral gastroenteritis was cured. Case 4 had diarrhea, along with her fever. Both the surgery and the radiational damage could weaken the defensive power of the intestine, which makes this kind of symptom likely to occur. Medical professionals should pay attention to a patient’s COVID-related atypical symptoms and laboratory test results to avoid misdiagnosis.

Also, as both radio- and chemo-therapy influences the leukocyte count, when a lower level of blood cell count appears, COVID-19 is not always initially suspected. The change caused by cancer therapy may even cover up the change by COVID-19. Case 1 was found to be cytopenic, with red blood cell and platelet count being rather low. In this situation, a seemingly normal leukocyte count of 6.67 × 10^9^/L might increase the difficulty of diagnosing COVID-19. Meanwhile, when case 2 was diagnosed, her leukocyte count was lower than normal, which conformed to diagnostic criteria. Additionally, Case 3, who had had chemotherapy eight times after an operation, would use long-acting G-CSF every time after treatment. Under the joint effect of G-CSF and COVID-19, when this case was diagnosed as having COVID-19, her leukocyte count was in a normal range, but lymphocyte was only 0.34 × 10^9^/L.

Finally, we focused on the influence of cancer treatment history in the treatment of COVID-19 patients. Cancer patients were in a low immunity state after radiotherapy, chemotherapy, and surgery for cancer, therefore, as for the treatment of these cases, in addition to symptomatic antiviral treatment, patients were given immune boosters and reagents that raised white blood cell count. Due to bone marrow suppression caused by radiotherapy and chemotherapy, case 1, case 2, and case 3 used agents for raising white blood cells during the treatment of COVID-19. All cases except for case 4 and case 6 applied immune enhancers (Table 1).

Apart from the side effects of radio-, chemo-therapy, and surgery, the immunotherapy and anti-PD-1 treatment of cancer patients might be beneficial to the treatment of COVID-19. At present, there were few reports about the effect of anti-PD-1 treatment on an infection of SARS-CoV-2. A study mentioned that viral infection could cause up-regulation in PD-1 to help virus immune escape (16), In our study, case 3 was infected with SARS-CoV-2 one month after receiving postoperative chemotherapy eight times and six rounds of anti-PD-1 therapy. The patient was in a poor condition and had symptoms of wheezing at the time of admission. Still, due to timely diagnosis and treatment, the patient’s condition improved after G-CSF and immunotherapy. After discharge, we conducted a telephone follow-up on the patient. So far, the patient’s COVID-19 had been cured with a positive antibody, and the condition of ovarian cancer is relatively stable. But, whether anti-PD-1 therapy and immunotherapy were beneficial to COVID-19’s treatment remains to be explored.

Therefore, the gynecological cancer patients became a more susceptible to contracting COVID-19, the history of cancer treatment made the diagnosis of COVID-19 difficult, which also affected the treatment of COVID-19, so we put forward some treatment suggestions for cancer patients and hospitals during the COVID-19 epidemic.

**FIGURE 3:**
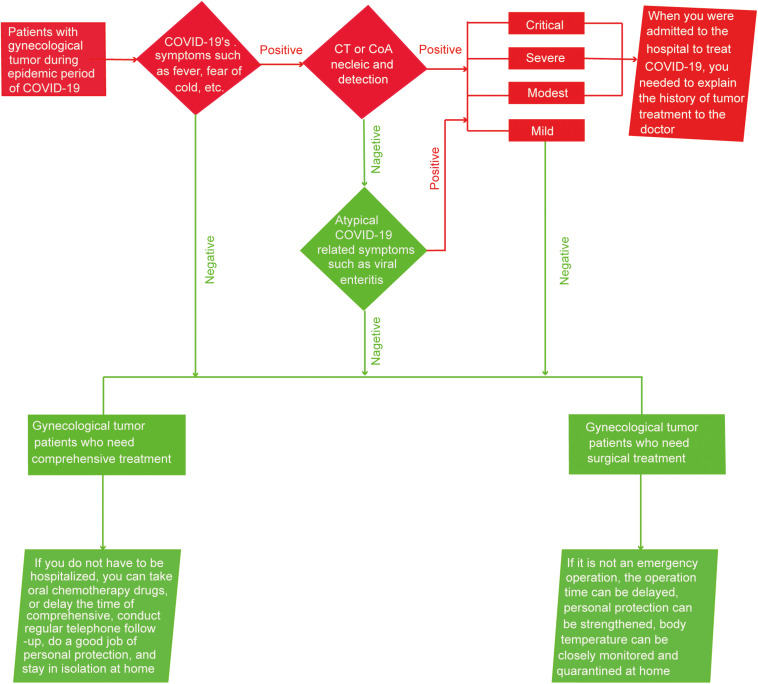
Diagram of patient visits in gynecological oncology department during the epidemic of COVID-19.

According to the current guidelines for the diagnosis and treatment of cancer patients, we made the following recommendations (8). If a patient with gynecological cancer had fever, chills, and other symptoms, a CT test and CoA nucleic acid test should be done to screen for COVID-19. If the patient’s CT showed COVID-19 related atypical pneumonia or the CoA nucleic acid test was negative, screening for other COVID-19 similar atypical symptoms, for example, viral enteritis, should become a priority. If the patient was diagnosed with a mild form of COVID-19 or did not have any signs of COVID-19, treatment should be divided into the following two situations based on the patient’s cancer condition. On the one hand, if the patient was currently diagnosed with cancer and surgery was needed but could be delayed, it was recommended to postpone, and if it was necessary to operate, COVID-19 patients should inform the attending doctor of their condition as soon as possible. On the other hand, if the patient was about to undergo or is currently undergoing radio-/chemo-therapy treatment and the condition was currently stable, it is recommended that home treatment measures such as oral chemotherapy drugs or delaying treatment time and conducting regular telephone follow-ups are put into effect (17). Additionally, patients infected with SARS-CoV-2 should carefully observe their condition and reexamine cancer at the same time. If the patient had modest, severe, or a critical case of COVID-19 and needed hospitalization, they should promptly inform the doctor of any tumor treatment history and closely observe the condition (Figure 3).

During the epidemic of COVID-19, except for the need for personal protection for patients, many studies now suggest that hospitals should also make good prevention strategies. Hospitals could manage patients according to mild, modest, severe, and critical cases, and set up isolation wards (14). Hospitals could also conduct regular telephone follow-ups for patients in need, moreover, patient channels and medical staff channels could be used to avoid casualties in medical staff (18).

## Data Availability Statement

The raw data supporting the conclusions of this article will be made available by the authors, without undue reservation.

## Ethics Statement

This study was approved by the Ethical Committee of Tongji Hospital of Tongji Medical College at Huazhong University of Science and Technology. Written informed consent was obtained from the individual(s) for the publication of any potentially identifiable images or data included in this article.

## Author Contributions

CL: conception and design. YH: collection and assembly of data. PW, CS, and GC: data analysis, interpretation, supervision, and modification. CL, YH, TQ, and EG: manuscript writing. All authors final approval of manuscript.

## Conflict of Interest

The authors declare that the research was conducted in the absence of any commercial or financial relationships that could be construed as a potential conflict of interest.
